# On Perceived Stress and Social Support: Depressive, Anxiety and Trauma-Related Symptoms in Arabic-Speaking Refugees in Jordan and Germany

**DOI:** 10.3389/fpubh.2020.00239

**Published:** 2020-06-30

**Authors:** Kerem Böge, Carine Karnouk, Eric Hahn, Zaynab Demir, Malek Bajbouj

**Affiliations:** Department of Psychiatry and Psychotherapy, Campus Benjamin Franklin, Charité – Universitätsmedizin Berlin, Corporate Member of Freie Universität Berlin, Berlin Institute of Health, Humboldt-Universität zu Berlin, Berlin, Germany

**Keywords:** mental health, perceived stress, social support, depression, anxiety, trauma, refugees

## Abstract

Current literature points toward several challenges in the access to sufficient and effective psychosocial care for Syrian refugees in host settings. This study is a comparative investigation into the relationship between “perceived social stress” and “perceived social support” on three of the most prevalent symptom dimensions in Syrian refugees across two host capitals, Berlin and Amman. Eighty nine Syrians refugees were recruited between January 2017 and March 2018. Participants were contacted through local institutions and organizations collaborating with the Charité—Universitätsmedizin Berlin. Assessments include the PHQ-9, GAD-7, HTQ, MSPSS, and PSS. Primary analyses consist of non- or parametric tests and multiple linear regression analyses. Subsample analyses showed relevant depressive, anxiety and trauma-related symptoms. Significant differences in PTSD symptoms (*p* < 0.04) were found. Participants reported high perceived stress and moderate to high social support. Linear regressions revealed that perceived stress had a significant negative effect (*p* < 0.01) on clinical outcomes in both subsamples. Perceived social support had a positive influence on depressive (*p* = 0.02) and PTSD symptoms (*p* = 0.04) for participants in Berlin. Analyses revealed significant positive effects of “significant others” (*p* = 0.05) on depressive- in Berlin and “family” (*p* = 0.03) support for PTSD symptoms in Amman. Study results show that levels of “perceived stress” appear to be the same across different host countries, whereas types of social support and their effect on mental health differ significantly depending on the host setting. Outcomes may guide future comparative study designs and investigations to promote well-being, integration, and the development of effective social support structures for the diverse needs of Arabic-speaking refugees.

## Introduction

The Syrian conflict, which is now approaching its eighth consecutive year, has forced more than 5.6 million of the country's citizens to take refuge in many corners of the world (1, 2). Countries in the Middle East and Europe were among the first to respond to the urgent plea for humanitarian aid and assistance. Of these nations, Germany and the Kingdom of Jordan have hosted a high number of Syrian refugees and asylum seekers (3). At first, Syrian citizens sought refuge in neighboring countries, but in response to the crisis, by the summer of 2015, Germany's open-door policy allowed Syrian citizens to request asylum and make Europe their new home (4).

Various barriers, which include cultural, linguistic, financial, as well as risks of discrimination, exploitation, and social isolation, have led to an inability to satisfy the basic needs of the Syrian refugee population (5). In turn, this has had a direct effect on mental health, prospects of integration and overall well-being (6). Recent studies have confirmed that the three most common psychiatric disorders observed in Syrian refugees are PTSD, depression and anxiety (1). Prevalence rates range between 20.5 and 35.7% for PTSD, 20 to 43.9% for depression (7, 8), and from 19.3 to 31.8% for anxiety disorders (9). Furthermore, loss and grief have been reported to be central themes (10). Other factors, such as the length of stay, living environment, uncertain residence status, acculturation processes, also seem to play a crucial role in the development of psychological distress (1).

In host environments, familiar sociocultural habits and routines are often disrupted (10). It has been reported that Syrian families often become estranged, report a loss of identity and a longing for home (5). According to Cohen and Syme (11), social support from family, friends and significant others (12) have been identified as protective factors “that aid in the maintenance of health as well as in disease recovery.” In Syrian host communities, basic community support groups, recreational spaces and development programs are scarce. Similarly, little attention is given to psychological and cognitive injuries, their consequences, and services to assist long-term recovery, despite available evidence psychosocial support as a coping resource and catalyst for positive change and well-being (13–15).

Combined, the Kingdom of Jordan and Germany have hosted more than 1 million Syrian refugees (3, 16). According to reports from 2019, there are a total of 664,330 registered Syrian refugees in Jordan (16) and there are about 646,665 asylum applicants in Germany since 2015 (17). Although both host countries are accommodating a large number of Syrian refugees, it seems like many sociodemographic variables influence refugee choices of a final destination (18). This choice is often informed by age, gender, education, marital status, national politics, and other factors. Although choices are often limited, some Syrian refugees choose to migrate to a neighboring country for reasons of “cultural proximity,” such a familiar language, similar religious values, national views and most importantly transferable skills (18). Some refugee communities also choose host countries, where they already have existing social ties and familial support networks. For young women, cultural norms and gender roles can play a role an important role in this choice (19), whereas this may be a different case for young men (20)—leading to diverse migration trends within one community. Furthermore, response to Syrian crisis, many organization and development programs have established mental health and psychosocial support activities, particularly in their capitals—Amman and Berlin (21).

Jordan is a relatively small, middle-income country with a climate that is influenced by ongoing political conflict, high poverty rates, and treatment gaps (22–24). Therefore, priority is given to the physical and basic needs of refugees (2, 25). In Jordan, the official national language is Arabic. Cultural customs and social fabrics are familiar to those of most Syrian refugees. According to a recent report (2), Jordan is currently considered the country with the highest number of NGOs operating in the MENA (Middle East and North Africa) region.

In contrast, Germany is a high-income country with developed structures (legal, medical, and educational), open recreational spaces and financial wealth. Nonetheless, Germany still seems to be facing ongoing challenges that are different from those of Jordan. These challenges are mostly related to linguistic, cultural and social barriers surrounding the integration and psychosocial support of refugee populations (5). In 2013, Charité Universitätsmedizin, Berlin was spearheading many initiatives in Jordan with its ChariteHelp4Syria project (CH4S) (5), yet despite increased knowledge and integrating best practice models, a treatment gap still remains.

Therefore, the present study aims to understand the relationship between “perceived stress” and “perceived social support” on the three most prevalent symptom dimensions (depressive-, PTSD-, and anxiety-related symptoms) observed in refugee populations residing in the capitals of two of the world's largest host countries for Syrian refugees—Amman and Berlin.

## Methods

### Participants and Procedure

Eighty nine Syrian refugees who resettled in either Berlin, Germany (*n* = 49) or Amman, Jordan (*n* = 40) were recruited between January 2017 and March 2018. In total, 89 participants were invited to take part in the study, all gave informed consent and none dropped out throughout the study process. In Berlin, participants were recruited at the central clearing clinic, an outpatient institution by Charité—Universitätsmedizin Berlin, specialized in offering psychiatric services for refugees and collaborates with multiple refugee camps and civic initiatives. In Amman, participants were recruited via the CharitéHelp4Syria project, a joint project of Charité and the German humanitarian non-governmental Organization “Help—Hilfe zur Selbsthilfe.”

For the study inclusion criteria were defined as (a) 18–65 years of age, (b) literate in Arabic language, and (c) having been exposed to the Syrian Civil War from 2011 onwards. Exclusion criteria included (a) lifetime diagnosis of psychotic disorder, bipolar disorder, personality disorder, (b) intellectual disability, (c) any mental disorder due to a general medical condition, and (d) current substance abuse.

An information sheet about study procedures was handed out by physicians. Participants were informed about the anonymity of data and their right to withdraw from the study at any time without giving a reason or the withdrawal having an impact on the services received by any governmental or non-governmental organizations. Ethics approval (EA4/067/10) was granted by the ethics review board of Charité—Universitätsmedizin Berlin according to the Declaration of Helsinki. All participants were provided with written informed consent and were financially reimbursed for their participation.

### Questionnaires

The self-rated Patient Health Questionnaire-9 (PHQ-9) (26) a validated Arabic instrument (27–29) which is used to assess the presence and severity of depressive symptoms. It includes a score range from 0 to 27. Responses for each of the nine items range from “0” (“not at all”) to “3” (“nearly every day”) with higher scores corresponding to higher symptom severity. In the present study, the PHQ-9 total score displayed good internal consistency (Cronbach's α = 0.85).

The Generalized Anxiety Disorder-7 (GAD-7) (30) is a self-reported screening instrument aiming to detect generalized anxiety symptoms and measure anxiety symptoms. It consists of seven items, which are scored on a four-point Likert-scale, ranging from “0” (“not at all”) to “3” (“nearly every day”). The validated Arabic version of the GAD-7 has been shown to have good psychometric validity (27, 29). In the current study, α was 0.86, indicating good internal consistency.

The Harvard Trauma Questionnaire (HTQ) (31) is a self-rated questionnaire assessing multiple facets of trauma experiences. The first part compromises of 42 items illustrating traumatic events, such as lack of food and clean water, torture, rape, and murder of family member or friend which are rated on a dichotomous scale: yes (1) and no (0). The second part consists of an open-ended question, in which participants can describe the most hurtful and terrifying. The third part encompasses 16 items, which aim to assess posttraumatic stress disorder symptoms (PTSD) severity. Responses are rated on a five-point Likert scale. Cut-off scores for current PTSD is set at >2.5 For the current study, the Arabic version of the HTQ was used, which has already been validated with refugees from Iraq and shown sufficient validity and a good test-retest reliability in previous studies (32, 33). Furthermore, part one and three showed good internal consistency with 0.89 and 0.87, respectively.

The self-report Multidimensional Scale of Perceived Social Support (12) is a brief questionnaire designed to measure perceptions of support from three main sources: (1) family, (2) friends, and (3) a significant other. The MSPSS comprises in total 12 items, subdivided into four items per subscale. Responses are given on a seven-point Likert scale. High scores resemble stronger perceived stronger support. The MSPSS was administered in Arabic language and its validation has shown good internal and test-retest reliability, good validity, and a fairly stable factorial structure (34). For the current study, α was 0.88, indicating good internal consistency.

The Perceived Stress Scale (PSS) (35) is a self-rated questionnaire developed to assess the degree to which situations in one's life are appraised as stressful. The PSS consists of ten items, is two-dimensional and includes positively and negatively phrased items. Participants give their responses on a five-point Likert scale. The Arabic version (36) of the administered PSS has good psychometric properties and displayed acceptable internal consistency (α) with 0.77 in the current study.

### Statistical Analysis

All data was collected, stored, and analyzed by using the Statistical Package for the Social Sciences (IBM, SPSS, Version 23), MacOS-X. Sociodemographic variables were descriptively represented using frequencies, percentages, means and standard deviations. Subsample analyses were performed to assess possible differences in clinical outcomes between both communities using non- or parametric tests, either one-tailed independent *t*-test or Mann-Whitney-U-Test. In a next step, regression analyses including non-standardized regression coefficient (B) and standardized regression coefficient (ß) were calculated using perceived social support and perceived stress as the independent variable and the clinical outcomes such as depressive-, anxiety-, and post-traumatic stress symptoms as the dependent variable. The level of significance was set at *p* < 0.05.

## Results

Demographic characteristics of the sample and both cohorts, Amman and Berlin, are summarized in Table 1. For the whole sample, participants were mainly female (53.9%), on average 33.9 years old, Syrian (96.6%), married (58.4%), not graduated from high school (39.3%), flew with their family (67.4%), escaped Syria for 42.71 months, and spent 39.15 months in German or Jordan, respectively. Furthermore, there are substantial differences between both cohorts as a majority in Berlin were male (59.2%) while in Amman female (70%). In Berlin, the age ranged between 18 and 40 years (93.8%) with an average of 30.00 (7.99) years while in Amman participants' age were rather balanced across years with an average of 38.9 (10.6). Furthermore, most participants who arrived in Berlin were single (53.1%) and flew alone (45.0 %) and were educated (81.6%) which stands in contrast to primarily married (82.5%) participants in Amman who escaped with their family (95%) and had not graduated from high school (65 %). Lastly, the departure from Syria was on average 29.2 (16.1) months ago for participants from Berlin and 59.3 (12.3) for Amman while time spent in the new country was 23.0 (11.6) and 58.9 (12.8) months indicating considerable difference between both cohorts, respectively.

**Table 1:** Sociodemographic characteristics of the survey sample and each subsample.

**Socio-demographic variables**	**Survey sample (*n* = 89)**	**Refugees living in Berlin (*n* = 49)**	**Refugees living in Amman (*n* = 40)**
**Gender [%]**			
Male	46.1	59.2	30.0
Female	53.9	40.8	70.0
**Age (range) [%]**			
18–30	41.6	57.1	22.5
31–40	36.0	36.7	35.0
41–50	12.4	0	27.5
51–60	10.1	6.1	15.0
**Nationality [%]**			
Syria	96.6	93.9	100.0
Iraq	1.1	2.0	0
Saudi Arabia	1.1	2.0	0
Palestine	1.1	2.0	0
**Marital status [%]**			
Single	36.9	53.1	15.0
Married	58.4	38.8	82.5
Divorced	5.6	8.2	2.5
**Educational status [%]**			
Not graduated high school	39.3	18.4	65.0
High school degree	28.1	34.7	20.0
Bachelor's degree	13.5	24.5	0
Master's degree	19.1	22.4	15.0
**Course of flight [%]**			
Alone	27.0	45.0	5.0
Family	67.4	44.8	95.0
Friends	5.6	10.2	0
**Months since departure from Syria [%]**			
0–24	30.3	53.1	2.5
25–48	24.8	34.7	12.5
49–72	39.3	12.2	72.5
73–96	5.6	0	12.5
**Months spent in Germany/Amman [%]**			
0–24	37.1	63.3	5.0
25–48	23.6	34.7	10.0
49–72	33.7	2.0	72.5
73–96	5.6	0	12.5

### Clinical Outcomes and Differences Between Both Communities

Concerning each subsample, results for participants from Berlin indicate relevant depressive—(8.31) and anxiety symptoms (7.89), which are at the threshold of mild to moderate symptom severity. With a cut-off score for current PTSD set at >2.5, participants illustrate post-traumatic stress symptoms bordering the diagnostic threshold (2.11). Furthermore, on average 15.98 of 43 items of the HTQ “after war” subscale was marked exhibiting relevant traumatic experiences. Participants from Berlin displayed high perceived stress (28.20) and were considered to have moderate, at the border to high, social support (4.69).

For participants from Amman, results demonstrated similar results with relevant depressive—(9.55) and anxiety symptoms (9.60), which are also at the cut-off threshold from mild to moderate symptom severity. Like the Berlin cohort, participants revealed post-traumatic stress symptoms at the diagnostic boarder (2.31) with 18.23 on average for the HTQ “after war” subscale. Perceived stress was high (26.91) and perceived social support at the border from moderate to high (5.09).

Statistical comparisons regarding clinical outcomes between subsamples demonstrated significant differences in post-traumatic stress symptoms (*p* < 0.04). Table 2 summarized all clinical outcomes including mean, standard deviation and *p*-values according to each subsample.

**Table 2:** Mean, standard deviation and *p*-values of clinical outcomes according to each subsample.

**Outcome Variable**	**Mean (SD)**	***p***
**PHQ-9^1^**		
Berlin (*n* = 49)	8.31 (4.64)	0.13^b^
Amman (*n* = 40)	9.55 (5.64)	
**PHQ-7^2^**		
Berlin (*n* = 49)	7.98 (4.74)	0.10^a^
Amman (*n* = 40)	9.60 (5.38)	
**HTQ after war^3^**		
Berlin (*n* = 49)	15.98 (6.99)	0.08^b^
Amman (*n* = 40)	18.23 (7.63)	
**HTQ PTSD^4^**		
Berlin (*n* = 49)	2.11 (0.59)	**0.04^b^**
Amman (*n* = 40)	2.31 (0.44)	
**PSS^5^**		
Berlin (*n* = 49)	28.20 (7.32)	0.21^b^
Amman (*n* = 40)	26.91 (7.23)	
**MSPSS^6^**		
Berlin (*n* = 49)	4.69 (1.34)	0.21^b^
Amman (*n* = 40)	5.09 (1.25)	

1 * = Cronbach's α = 0.85*;

2 * = Cronbach's α = 0.86*;

3 * = Cronbach's α = 0.89*;

4 * = Cronbach's α = 0.87*;

5 * = Cronbach's α = 0.77*;

6 * = Cronbach's α = 0.88*;

a * = Mann-Whitney-U Test*;

b * = independent samples t-test; α = 0.05 (one-tailed)*.

*Significant p-values < 0.05 are marked in bold*.

### Regression Analysis

To analyse the impact of perceived social support and perceived stress on symptoms of depression, anxiety and post-traumatic stress, multiple linear regression analyses were performed. Overall, regression analyses revealed that perceived stress had a significant negative effect (*p* < 0.01) on all three clinical outcomes in Berlin as well as Amman. However, regression analyses concerning the influence of perceived social support on depressive, anxiety, and post-traumatic stress symptoms showed significant positive effects for two clinical outcomes in Berlin but not in Amman. Here, results indicate that perceived social support had a positive influence on depressive- (β = −0.065; *p* = 0.02) and post-traumatic stress symptoms (β = −0.009; *p* = 0.04) for participants in Berlin. On a subscale level, analyses displayed a significant positive effect of “significant other” (β = −0.118; *p* = 0.05) on depressive- in Berlin and “family” (β = −0.029; *p* = 0.03) on post-traumatic stress symptoms in Amman. A summary of the primary regression analyses, including on-standardized regression coefficient (*B*) and standardized regression coefficient (ß) and *p*-values (*p*) for each subsample, are depicted in Table 3.

**Table 3:** Regression analysis for perceived stress and social support on depression, anxiety, and post-traumatic stress symptoms in Berlin and Amman with beta scores, standardized beta values, and their significance values.

	**Berlin**			**Amman**		
**Questionnaire**	**b**	**ß**	***p***	**b**	**ß**	***p***
**PHQ-9**						
MSPSS	−0.065	−0.240	**0.02**	−0.018	−0.048	0.36
PSS	0.325	0.595	**<0.01**	0.571	0.696	**<0.01**
**PHQ-7**						
MSPSS	−0.042	−0.143	0.10	−0.063	−0.178	0.12
PSS	0.414	0.642	**<0.01**	0.428	0.547	**<0.01**
**PTSD**						
MSPSS	−0.009	−0.230	**0.04**	−0.001	−0.030	0.43
PSS	0.033	0.412	**<0.01**	0.034	0.535	**<0.01**

*Significant p-values < 0.05 are marked in bold*.

## Discussion

The present study aimed to explore “perceived stress” and “perceived social support” on three of the most prevalent symptom dimensions including depressive-, PTSD- and anxiety symptoms in Syrian refugees (1) in both host capitals, Berlin and Amman. Similar to other studies (1), the main findings of this study revealed that perceived stress has a significant negative effect on all three clinical outcomes in both cohorts. Moreover, perceived social support showed positive effects for *only* depressive—and PTSD symptoms in the Berlin sample, but not for Amman. There were no associations observed between perceived social support and anxiety symptoms in both samples. When analyzing the subscales of “perceived social support”, only two types of social support had a positive influence on the participants' mental health. In the Berlin cohort, “perceived social support” from a “significant other” had a positive effect on depressive symptoms, whereas, in the Amman sample, support from a “family member” had a positive effect on trauma-related symptoms.

According to global trends in forced displacements, most refugee communities remain close to their homeland, while only a small number of individuals move to more distant and remote host countries. While most arrivals in Jordan were documented between 2012 and 2015 (37), Germany's open door policy gave access to asylum seekers mostly in the summer of 2015 (4). This timeline thus reflects a realistic representation of why participants reported having stayed for longer periods in Amman. Official data from census also confirm that over two-thirds (about 69.2%) of asylum seeking applicants in Germany are also males (20) reflecting the significantly higher prevalence of educated, single males in the Berlin sample.

Germany offers newly arriving refugees integration- and language courses, as well as professional development opportunities (38). In contrast, the Kingdom of Jordan offers proximity to home, a similar language, cultural norms and more easily transferable skills. Young adult males may be fulfilling their familial duties of “scouting the route” for other “more vulnerable” family members that are yet to follow (possibly through reunification programs). However, for young Syrian refugee women, a close tie to gender and cultural norms, especially with regards to marital prospects and proximity to family may take precedence over professional or economic goals (5, 19) The motivations to favor some host countries over others may have led to the heterogeneity of sociodemographic variables within our samples, making a sound methodological comparison practically impossible. In contrast, one of the major strengths of this study is its ecological validity, in which real-life circumstances and similarities are clearly noticeable in our cohorts. It is, thus, important to interpret the study's data cautiously without making claims of inferences or causality. Nonetheless, results from such studies may help policy makers in the development and implementation of more formal and visible social support structures. Findings may also influence new treatment models that are more suitable for this population's needs and offer compelling evidence in support of new scalable peer-to-peer intervention efforts, such as the STRENGTHS project (39), and other hybrid stepped-care models, such as MEHIRA (Mental Health of Refugees and Asylum Seekers (40).

A significant strength of this study is that the results give first insights into the types of social support that have shown to have a significant positive effect on Syrian refugee mental health. So far, the relationship between mental health and social support has been under-investigated in this vulnerable population, although there has been evidence proving the general benefits of social support on mental and physical well-being (11, 41, 42). In the Berlin cohort, it seems like support offered by a “significant other” had a positive influence on depressive symptoms, whereas “family” support seemed to alleviate trauma-related symptoms in the Amman sample. The Syrian culture is known for its rich cultural customs and traditions, as well as strong familial relationships and social fabrics (5). Therefore, family separation undoubtedly leads to increased feelings of emotional distress (5, 43). Because most of the Berlin sample is made up of single males, it may be possible that, as a coping mechanism, this cohort relies on social support from “significant other” as a substitute for the absent family. In contrast, within the Amman sample, which is made up of mostly Syrian women, relying on the family unit may reflect traditional gender roles. Investigating further aspects were not within the scope of the present paper, but it is imperative that future studies also focus “within-group” differences in experiences and perceptions of stress and social support needs of refugee communities.

Regarding the limitations of the study, all variables were assessed with subjective, self-report questionnaires, however reports of past experiences may be prone to reporting bias (44). Moreover, no psychiatric standardized interview was conducted to assess diagnostic criteria. Therefore, clinical outcomes only display symptom dimensions. Furthermore, the research is cross-sectional limiting any conclusions regarding causality and generalizability. Due to limited resources, the role of some factors such as resilience, education, socioeconomic background, were beyond the scope of this paper. Such analyses may be useful to follow up on in future research to make meaningful associations.

In conclusion, the present study gives the first comparative insights into the relationship between “perceived social stress” and “perceived social support” on three most prevalent symptom dimensions in Syrian refugees in both Germany and Jordan's capitals—Berlin and Amman. Overall, results show that “perceived stress” levels are the same across different host countries; however, types of social support and their effect on symptoms differ significantly depending on the host setting.

## Data Availability Statement

The datasets generated for this study are available on request to the corresponding author.

## Ethics Statement

The studies involving human participants were reviewed and approved by Charité - Universitätsmedizin Berlin, Ethics Committee. The patients/participants provided their written informed consent to participate in this study.

## Author Contributions

KB, CK, EH, ZD, and MB contributed to the study conception and research design. ZD and MB led all aspects concerning data recruitment and assessment. KB, CK, EH, and MB contributed to the drafting of the manuscript. KB conducted the data analysis while ZD prepared the data sheets. All authors commented and contributed to the final manuscript and have seen and given final approval of the version to be published.

## Conflict of Interest

The authors declare that the research was conducted in the absence of any commercial or financial relationships that could be construed as a potential conflict of interest.
